# Supplementary material for: Comparative genomics provides new insights into the diversity, physiology, and sexuality of the only industrially exploited tremellomycete: Phaffia rhodozyma
Source: BMC Genomics. 2016 Nov 9;17:901. doi: 10.1186/s12864-016-3244-7 (PMC5103461; doi:10.1186/s12864-016-3244-7)
Supplement: Additional file 6: — List of orphan genes with links to PFAM (related to Additional file 1: Table S1). (ZIP 1428 kb) [file 12864_2016_3244_MOESM6_ESM.zip › PFAM_UNIQUE/G01289_P.html]

#                                                                            --- full sequence --- -------------- this domain -------------   hmm coord   ali coord   env coord
  
# target name        accession   tlen query name           accession   qlen   E-value  score  bias   #  of  c-Evalue  i-Evalue  score  bias  from    to  from    to  from    to  acc description of target
  
#------------------- ---------- ----- -------------------- ---------- ----- --------- ------ ----- --- --- --------- --------- ------ ----- ----- ----- ----- ----- ----- ----- ---- ---------------------
  
G01289\_P             -            590 Adeno\_hexon          PF01065.14   495       1.4    6.3   7.7   1   1   0.00088       2.6    5.4   7.7    98   211   266   381   238   403 0.70 - Hexon, adenovirus major coat protein, N-terminal domain  
G01289\_P             -            590 Cytomega\_UL20A       PF05984.7    100       3.7    6.5  13.4   1   2     0.043   2.6e+02    0.6   0.0     1    32     1    31     1    53 0.76 - Cytomegalovirus UL20A protein  
G01289\_P             -            590 Cytomega\_UL20A       PF05984.7    100       3.7    6.5  13.4   2   2     0.003        18    4.3  10.8    14    90   281   357   275   366 0.84 - Cytomegalovirus UL20A protein  
G01289\_P             -            590 Daxx                 PF03344.10   713      0.27    8.3   6.1   1   1    0.0061       0.5    7.4   6.1   419   507   278   366   210   429 0.63 - Daxx Family  
G01289\_P             -            590 DHHW                 PF14286.1    378       8.7    4.2   8.8   1   1     0.011        22    2.8   8.8    57   158   266   371   250   397 0.47 - DHHW protein  
G01289\_P             -            590 IKI3                 PF04762.7    928    0.0061   13.1   9.8   1   1   9.8e-06    0.0083   12.6   9.8   393   627   135   382   108   408 0.78 - IKI3 family  
G01289\_P             -            590 MIP-T3               PF10243.4    539      0.13    9.3   5.1   1   1    0.0016       0.2    8.7   5.1   198   320   275   486   238   519 0.66 - Microtubule-binding protein MIP-T3  
G01289\_P             -            590 Mitofilin            PF09731.4    582      0.57    7.4   6.9   1   1    0.0065      0.78    7.0   6.9    75   167   276   367   207   497 0.61 - Mitochondrial inner membrane protein  
G01289\_P             -            590 SPATIAL              PF15256.1    196       1.3    7.7   7.6   1   1    0.0026       3.9    6.2   7.6    35   129   287   377   214   383 0.79 - SPATIAL
